# The Role of Nrf2 in Hearing Loss

**DOI:** 10.3389/fphar.2021.620921

**Published:** 2021-04-12

**Authors:** Dafei Li, Haiyan Zhao, Zhong-Kai Cui, Guangyong Tian

**Affiliations:** ^1^ Department of Otorhinolaryngology-Head and Neck Surgery, The Third Affiliated Hospital of Southern Medical University, Guangzhou, China; ^2^ Guangdong Provincial Key Laboratory of Bone and Joint Degeneration Diseases, Guangzhou, China; ^3^ Department of Cell Biology, School of Basic Medical Sciences, Southern Medical University, Guangzhou, China

**Keywords:** antioxidant, NIHL, ROS, Nrf2, ARHL, ototoxic hearing loss

## Abstract

Hearing loss is a major unresolved problem in the world, which has brought a heavy burden to society, economy, and families. Hair cell damage and loss mediated by oxidative stress are considered to be important causes of hearing loss. The nuclear factor erythroid 2–related factor 2 (Nrf2) is a major regulator of antioxidant capacity and is involved in the occurrence and development of a series of toxic and chronic diseases associated with oxidative stress. In recent years, studies on the correlation between hearing loss and Nrf2 target have continuously broadened our knowledge, and Nrf2 has become a new strategic target for the development and reuse of hearing protection drugs. This review summarized the correlation of Nrf2 in various types of hearing loss, and the role of drugs in hearing protection through Nrf2 from the literature.

## Introduction

Hearing loss is a great challenge for the physical health and communication, which affects 6–8% of the population all over the world. In 1985, only 42 million people suffered from hearing loss, while recent updated statistics revealed that the number has rapidly increased to nearly 500 million (Wilson et al., 2017). According to the latest estimates from the World Health Organization, unresolved hearing loss costs about $750 billion a year globally (Marx et al., 2018). The etiology of hearing loss is complex and diverse, and it is currently acknowledged that viral infection, microcirculation disorder, autoimmunity, gene mutation, membranous labyrinth rupture, congenital ear malformation, noise and drug toxicity are involved in the occurrence and development of hearing loss (Cunningham and Tucci, 2017). Sensorineural hearing loss (SNHL) is the most common type of hearing loss, including noise induced hearing loss (NIHL), age-related hearing loss (ARHL) and ototoxic hearing loss. Within three subtypes of human hearing loss, the accumulation of reactive oxygen species (ROS) in hair cells can be observed by pathological examinations. Hair cell damage and loss mediated by oxidative stress are considered to be important causes of hearing loss. Understanding the changes in the expression of antioxidant factors in hearing loss is helpful for the prevention and treatment of hearing loss.

Nuclear factor erythroid-2 related factor 2 (Nrf2) as an important transcription factor in regulating oxidative stress response of cells, plays an important role in maintaining cell redox homeostasis (Ma, 2013; Cuadrado et al., 2018). Nrf2 reduces the cell damage caused by oxidative stress and maintains the dynamic balance of the systematic redox by inducing and regulating the expression of various antioxidant factors. Numerous studies have shown that Nrf2 is a major regulator of a variety of cellular protective responses and a key molecular node in a specific disease group, providing a new strategic target for drug development and reuse (Cuadrado et al., 2019). This review systematically summarized the correlation studies of Nrf2 in hearing loss, providing ideas for the prevention and treatment of hearing loss with Nrf2 as the target. At present, the research on the treatment for hearing loss with drugs targeting Nrf2 is progressing gradually, and has only achieved satisfied results in cell and animal experiments. Drugs reduce oxidative stress damage in hair cells by promoting the intracellular translocation of Nrf2 and enhancing the transcription of its downstream antioxidant factors. However, there is still a long way to go in the clinical application of Nrf2 activator in the treatment for hearing loss.

In this review, we delineated the structure, function, and localization of Nrf2 in the animal and human cochlea. Moreover, the correlation summary of Nrf2 is specially focused on the three subtypes of hearing loss, namely NIHL, ARHL, and ototoxic hearing loss. In addition, the current development of drug research on Nrf2 for the prevention and treatment of hearing loss was summarized, and we provided our perspectives on Nrf2 as a strategic target for the prevention and treatment of hearing loss.

## Structure and Function of Nrf2

Nrf2 is a transcription factor encoded by NFE2L2 gene (Sykiotis and Bohmann, 2010). In both humans and common house mice, the NFE2L2 gene is located on chromosome 2. Nrf2 has a highly conservative basic region-the Leucine Zipper (bZIP) structure, which contains seven domains, respectively named Neh1 -- Neh7 (Nrf2-ech Homology) (Jaramillo and Zhang, 2013). The Neh1 region contains a bZIP structure. Small Maf proteins (sMafs) are bZIP-type transcription factors that can bine to DNA and regulate gene regulation. Through the leucine zipper structures, sMafs form homodimers by themselves (Kataoka et al., 1995) and heterodimers with other specific bZIP transcription factors, including the Cap 'n' collar (CNC) proteins (p45 NF-E2, Nrf1, Nrf2 and Nrf3) (Igarashi et al., 1994; Kobayashi et al., 1999) and Bach family (Bach1 and Bach2) (Oyake et al., 1996). sMafs, as the key regulatory center of CNC-sMaf transcription factor network, play an important role in various biological pathways (Katsuoka and Yamamoto, 2016). When Nrf2 is translocated into the nucleus, bZIP forms heterodimers with sMafs proteins in the nucleus, which enable Nrf2 to identify antioxidant response element (ARE) (nucleotide sequence is 5’-(G/A)TGA (G/C)nnnGC (G/A)-3’(n represents any type of nucleotide), and thus initiate downstream related gene transcription. Both DLG (aspartate, leucine, and glycine) and ETGE (glutamate, threonine, glycine, and glutamate) are important conserved regions that bind to Kelch-like ECH-associating protein 1 (Keap1), and the Neh2 region stabilizes the presence of Nrf2 in the cytoplasm through the interaction between DLG, ETGE and Keap1. Located at the C terminal of Nrf2, Neh3 can bind to CHD6 (a Chromo-ATPase/helicase DNA binding protein) and promote the regulatory effect of ARE on the transcription of related genes. Neh4 and Neh5 are structural domains involved in initiating downstream gene transcription. Only when Nrf2 is translocated into the nucleus and binds to ARE in the form of Nrf2-Maf, thereafter combines with Neh4, Neh5 and CREB, etc., the transcription process can be activated. Neh6 is a non-KEAP1-dependent regulatory region degraded by Nrf2 and contains abundant serine. Neh7 region mediates the physical binding of Nrf2 to retinoic acid X receptor, which can inhibit the transcriptional activity of Nrf2 (Wang et al., 2013).

Nrf2 activation is associated with two regulators, Keap1 and Cullin3 (Cul3) (Williamson et al., 2012; Cuadrado et al., 2019). The DLG and ETGE regions of Neh2 have different binding affinity with Keap1. Compared with the ETGE region, the binding of DLG region to Keap1 is weaker, and the bound DLG-Keap1 is likely to separate due to the alteration of Keap1 conformation. Under normal physiological conditions, Nrf2 is anchored in the cytoplasm after binding to Keap1. As the substrate of Cul3-dependent E3 ubiquitin ligase complex, Keap1 binds to Nrf2, causing ubiquitination of Nrf2 and its rapid degradation by proteases. However, in the state of oxidative stress, the binding between Keap1 and DLG disconnects, and the ubiquitination of Nrf2 is blocked. Therefore, the newly synthesized Nrf2 can be transferred and accumulated into the nucleus (Itoh et al., 2004; Jung and Kwak, 2010; Baird and Dinkova-Kostova, 2011). After the dissociation of Nrf2 and Keap1, Nrf2 rapidly translocates into the nucleus, which forms an isodimer with small Maf proteins, binds ARE, and thereafter initiates the transcription of downstream antioxidant enzyme genes regulated by Nrf2. Common downstream gene products include nicotinamide adenine dinucleotide phosphate (NADPH), quinone oxidoreductase 1 (NQO1), glutathione-S-transferases (GSTs), glutamate cysteine ligase catalytic (GCLC) and heme oxygenase-1 (HO-1), etc. In addition, Nrf2 also plays a key role in regulating the redox system of L-γ-glutamyl-cysteinyl-glycine (GSH). Nrf2 regulates GSH biosynthesis by regulating the expression of the rate-limiting enzyme γ-glutamyl cysteine synthetase (γ-GCS) (Zhu et al., 2008).

In animals, Nrf2 is closely related to various diseases by regulating oxidative stress response (Cuadrado et al., 2019). Drug enhancement of the electrophilic counterattack organized by Nrf2 may be used as a strategy for chemoprophylaxis of cancer (Prestera et al., 1993; Satoh et al., 2013; Panieri et al., 2020), and has been well validated in various experimental cancer models in mice (Milkovic et al., 2017). Moreover, Nrf2 has a broad protective effect on lung diseases such as asthma, neurodegenerative diseases such as Parkinson's disease, inflammatory diseases such as inflammatory bowel disease, liver damage, atherosclerosis, insulin resistance and so on (Sykiotis and Bohmann, 2010; Cuadrado et al., 2019).

Unlike animal studies, the role of Nrf2 in human diseases is much less reported. Research on the role of Nrf2 in human diseases mainly focuses on cancer (Liu et al., 2020), respiratory diseases (Cuadrado et al., 2020), neurodegeneration (Fao et al., 2019). Restricted by ethics, DNA polymorphism analysis and changes in mRNA and protein content are the main means to study the protective effect of Nrf2 on a variety of human diseases. More importantly, studies on the role of Nrf2 in hearing loss are scarcely present in clinical trials.

## Localization of Nrf2 in the Inner Ear

Nrf2 is widely expressed in tissues including brain, retina and inner ear (Hoshino et al., 2011; Zhong et al., 2013; Yamazaki et al., 2015). Nrf2-immunoreactivity (IR) is found mainly in the human Corti organ at the apical, medial and basal region (Hosokawa et al., 2018). In humans, Nrf2 locates in the cytoplasm and nuclei of Corti organ, including inner hair cells (IHC), outer hair cells (OHC) and supporting cells (Deiters and Hensen cell) (Hosokawa et al., 2018). In cells of the spiral prominence and spiral limbus, Nrf2 is found to be immunoreactive, but hardly in spiral ganglion cells (Hosokawa et al., 2018). In addition, Nrf2 immunoreactivity is not found in other cochlea structures, such as stria vascularis, spiral ligaments and Reissner membrane (Hosokawa et al., 2018). The localization of Nrf2 in human inner ear in normal or pathological conditions remains to be further verified due to ethical issues, as well as differences in sampling time and methods of human inner ear.

**FIGURE 1:**
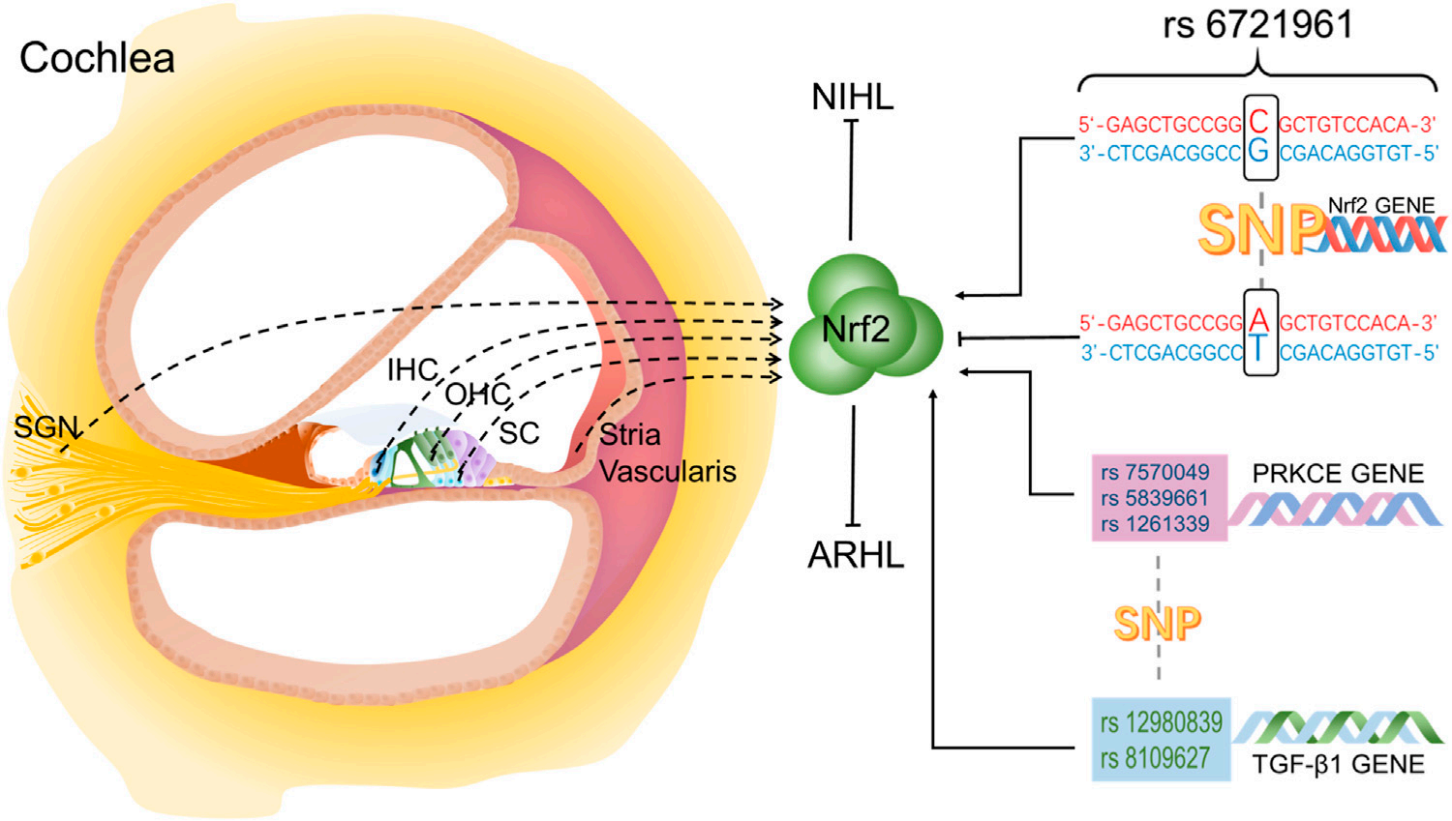
Localization of Nrf2 in the inner ear and the effect of SNPs on Nrf2. Nrf2 is located in IHC, OHC, SC, SGN and stria vasculatures in cochlea.

In contrast, animal experiments involving the localization of Nrf2 in the inner ear are well established and reported. Different from the normal human cochlea, in the normal rat cochlea, Nrf2 immune response is only detected in the cytoplasm of hair cells, support cells and spiral ganglion neurons (SGNs), and no immune response occurs in the nucleus (Hosokawa et al., 2018). However, under pathological conditions, Nrf2 can be observed in the cytoplasm and nucleus of the IHC, OHC and support cells in the Corti organ (Fetoni et al., 2015b; Hosokawa et al., 2018). Nrf2 is also reported in the cytoplasm and nucleus of stria vascularis and SGNs. In addition, after the application of antioxidants and Nrf2 activators or the stimulation of noise and ototoxic drugs, immunofluorescence staining showed that the expression of Nrf2 in hair cells increased in cytoplasm and translocated into nucleus (Fetoni et al., 2015b; So et al., 2006). Altogether, Nrf2 is clearly present in the inner ear (Figure 1), and plays an important role in different cells. The distribution of Nrf2 in cochlea may be different between humans and animals or different species of animals. There is a single nucleotide polymorphism (SNP) (rs6721961) in the promoter region of human Nrf2 gene, and in the presence of T allele, human hearing seems to be more vulnerable to occupational noise exposure (Honkura et al., 2016). Moreover, three SNPs of the Protein Kinase C Epsilon (PRKCE) gene (rs12613391, rs5839661, rs7570049) and two SNPs of the transforming growth factor-β1 (TGF-β1) gene (rs12980839, rs8109627) have been associated with ARHL (Fetoni et al., 2018).

## Correlation Between Hearing Loss and Nrf2

Peripheral hearing loss is usually divided into conductive hearing loss, sensorineural hearing loss or mixed hearing loss with both conductive and sensorineural hearing loss (Cunningham and Tucci, 2017). Most patients suffering from conductive hearing loss usually return to normal after drug or surgical intervention. Unfortunately, medical treatments for sensorineural hearing loss and mixed hearing loss typically received unsatisfied results, because of the damaged sensory hair cells and/or auditory neurons. Sensorineural hearing loss is the clinically common type of hearing loss, mainly resulting from aging, gene mutation, chronic diseases, noise and ototoxic drugs, etc. NIHL, ARHL and ototoxic hearing loss are the three subtypes of sensorineural hearing loss (Fujimoto and Yamasoba, 2019). All three subtypes of hearing loss can be pathologically observed with damage to cochlear hair cells and/or damage to auditory neural pathways. Most importantly, oxidative stress and the imbalance of redox homeostasis caused by ROS are important factors for cochlear injury (Gentilin et al., 2019; Rousset et al., 2020; Wu F. et al., 2020). As Nrf2 is an important regulator of antioxidant function, the current studies on the role of Nrf2 in hearing loss on NIHL, ARHL and ototoxic hearing loss are summarized.

### Correlation Between Nrf2 and NIHL

Oxidative stress caused by auditory trauma is the main pathway of cochlear injury (Henderson et al., 2006). There is an increase of ROS in the lymphatic fluid of the cochlea, HCs and the stria vascularis after noise exposure (Ohlemiller et al., 1999; Yuan et al., 2015). During noise exposure, blood circulation in the cochlea is impaired due to the contraction of capillaries in the cochlea and vestibular tissues (Quirk et al., 1992; Quirk and Seidman, 1995). After noise exposure, cochlear blood flow gradually recovers, causing cochlear ischemia reperfusion injury (Yamane et al., 1991), and increasing the production of ROS and reactive nitrogen species (RNS) (Yamashita et al., 2005; Fetoni et al., 2019; Wu et al., 2020). In addition, oxidative stress caused by noise exposure over stimulates mitochondria, leading to an increase in mitochondrial aerobic respiration, which in turn leads to the production of a large number of ROS (Maulucci et al., 2014). With the accumulation of ROS, the innate antioxidant defense ability of the cochlea is eventually exceeded, resulting in the damage of Corti organ and the apoptosis of hair cells, ultimately leading to hearing loss.

**FIGURE 2:**
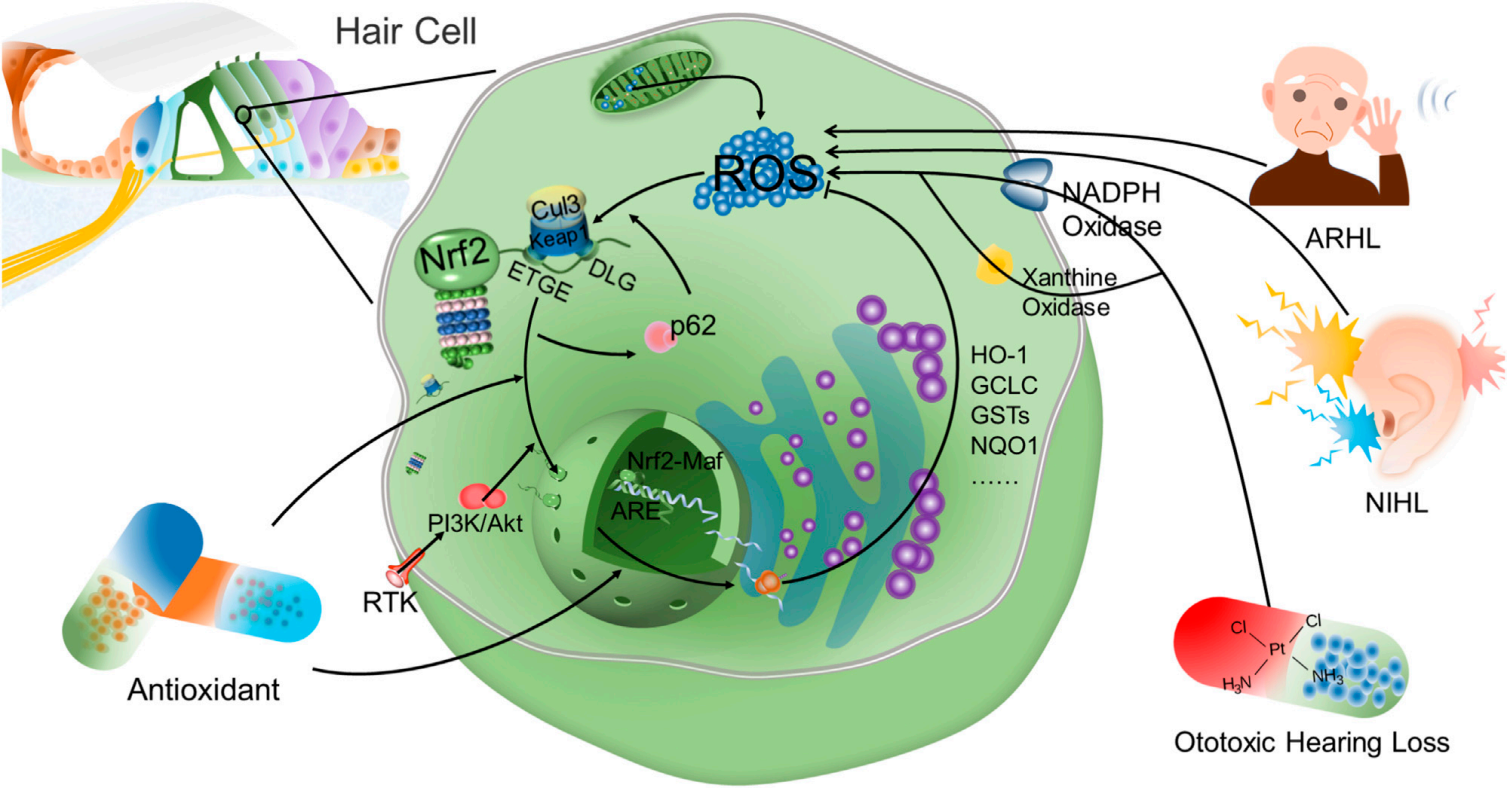
Nrf2 is an important factor to reduce oxidative stress injury of hair cells. Under normal conditions, Nrf2 is anchored in the cytoplasm by Keap1 and is rapidly degraded by ubiquitination. However, Nrf2 is disconnected from Keap1 and translocates into the nucleus after the action of oxidative stress. Antioxidant drugs have been found to promote the intracellular translocation of Nrf2 and the transcription of downstream antioxidant products after Nrf2 bound to ARE. After activation of Keap1-Nrf2-ARE signaling axis, antioxidant factors such as HO-1 can reduce the intracellular ROS level mediated by NIHL, ARHL and ototoxic hearing loss, thereby protecting hair cells and reducing hearing loss. Moreover, there is a positive feedback between p62-mediated autophagy and Nrf2. p62 destroyed the connection between Keap1 and Nrf2 to promote the accumulation of Nrf2 and nuclear translocation. Concurrently, increased Nrf2 promotes p62 expression. In addition, PI3K-Akt signaling pathway is also involved in the intracellular translocation of Nrf2 in hair cells. However, in the study on hearing loss, the relationship between other signaling pathways and Nrf2 expression is still not fully understood.

Nrf2 is an important factor to resist oxidative stress injury in NIHL (Figure 2). After exposure to noise, Nrf2 in cytoplasm increases slightly, however, it is not able to prevent the damage of reactive oxygen species to hair cells, and noise exposure has no significant influence on the expression of Nrf2 target genes (Honkura et al., 2016). By contrast, after the application of antioxidant and Nrf2 inducer, Nrf2 expression in cytoplasm and intracellular translocation are significantly increased. Confocal analysis has further confirmed that Nrf2 transports from cytoplasm into nucleus (Fetoni et al., 2015), thus binding to intracellular antioxidant reaction elements and activating transcription of downstream antioxidant factors. The regulation and expression of HO-1 in hair cells and helical ganglion neurons in the same area is in parallel with the translocation of Nrf2 from cytoplasm into nucleus, and the increase of GSH adduct is observed at the same time. This suggests, in NIHL, Nrf2 intracellular translocation activates Nrf2-ARE signaling pathway, and through enhancing the expression of HO-1 and superoxide dismutases (SODs) in cochlear hair cells and spiral ganglion, thereby reducing hearing loss caused by noise to rats, reducing auditory brainstem response (ABR) threshold drift, and promoting hair cell survival. The up-regulated HO-1 induced by Nrf2 affects the survival of hair cells that amplify and transmit acoustic signals, and indirectly affects the signal transmission from primary afferent neurons to the central auditory pathway (Fetoni et al., 2015). Moreover, the antioxidant stress injury induced by Nrf2 is time-correlated. In studies on the resistance to NIHL with rosmarinic acid (RA, a natural antioxidant), the expression of HO-1 and SODs increases continuously over time after the Nrf2-ARE signaling pathway is induced and activated by RA (Fetoni et al., 2015).

Nrf2 deficiency aggravates NIHL. ABR threshold movement after 7 days of noise exposure is significantly different in mice with and without Nrf2 gene, and Nrf2^−/−^ mice are more susceptible to noise. In Nrf2^−/−^ mice, the expression levels of Nrf2 target genes (NQO1, HO-1, GCLC, GCLM, and Txnrd1) significantly decrease after oxidative stress induction, and the reduction of GSH is also demonstrated (Honkura et al., 2016). It is worth mentioning that there is an SNP (rs 6721961) in the promoter region of human Nrf2 gene. In the rs 6721961 genotype, G and T alleles lead to high and low expression of Nrf2 mRNA, respectively (Yamamoto et al., 2004; Suzuki et al., 2013). The study found that in the presence of the T allele, human hearing threshold at 4 kHz seemed more susceptible to increased exposure to occupational noise (Honkura et al., 2016). The study also pointed out that Nrf2 must be activated before noise exposure to achieve significant hearing improvement. When exposed to noise, sufficient antioxidant capacity mediated by Nrf2 is essential to inhibit the progression of NIHL.

### Correlation Between Nrf2 and ARHL

ARHL and presbycusis are also important health problems lowering the quality of life (Figure 2). ARHL is characterized by age-related hearing loss and is thought to be associated with loss of SGNs and sensory hair cells in the inner ear cochlea (Yamamoto et al., 2004; Liu and Yan, 2007). Chronic inflammation and programmed cell death in cochlea caused by changes in antioxidant enzyme levels, oxidative stress and decreased activity of mitochondrial respiratory chain complexes I, II, IV (NADH oxidase, succinate dehydrogenase, and cytochrome c oxidase, respectively) are important molecular mechanisms for premature ARHL (Someya et al., 2010; Someya and Prolla, 2010; Menardo et al., 2012; Rousset et al., 2020). Oxidative stress injury of cochlea is considered to be the most important cause of ARHL (Yamasoba et al., 2013).

Nrf2 deficiency or decreased expression is involved in the occurrence and development of ARHL. Tubby strain of obesity mice develops progressive hearing loss due to gene mutations. This mutation causes OHC loss in the hook area of cochlea by 1 month of age and the damaged area expands to the apical turn by about 6 months of age. In addition, damage to inner hair cells was also observed in the hook area by about 6 months of age in the tubby mouse model (Ohlemiller et al., 1995). In homozygous mutant tubby mice (tub/tub), Nrf2 expression is significantly reduced, and ROS accumulation in hair cells leads to cell death (Kong et al., 2009). Compared with wild-type mice matched with the same age, Nrf2 gene completely knockout mice show a greater degree of hearing loss with age, and a faster loss rate of HCs and SGNs in cochlea (Hoshino et al., 2011). Some studies have found that the gap junction protein beta-2 (GJB2) gene, also known as connexin 26 (Cx26), and 35delG heterozygous (human) carriers have hearing loss in excess of 4 kHz frequency (Franze et al., 2005). In GJB2^+/−^ mice, the function of the Cx26 is completely lost, which shows that the expression of Nrf2 is significantly reduced and accelerates the occurrence of ARHL (Fetoni et al., 2018).

SNPs in antioxidant genes are also involved in hearing loss by influencing Nrf2. PRKCE and TGF-β1 are two members of Nrf2 signaling pathway. Three SNPs of PRKCE gene (rs 12613391, rs 5839661, rs7570049) and two SNPs of TGF-β1 gene (rs 12980839, rs 8109627) are closely related to ARHL in a matched and control study of healthy volunteers and elderly deafness patients (Fetoni et al., 2018) (Figure 1). In GJB2^−/−^ mice, down-regulation of TGF-β1 is at least partially protective against hearing loss (Fetoni et al., 2018).

### Correlation Between Nrf2 and Ototoxic Hearing Loss

Platinum anticancer drugs and aminoglycoside drugs are the main clinical ototoxic drugs (Brock et al., 2018; Kros and Steyger 2019; Zada et al., 2020). Cisplatin, as a highly effective chemotherapy drug, exhibits a good effect on tumors in children and adults, especially on head and neck squamous cell carcinoma. Irreversible sensorineural deafness is one of the main side effects from cisplatin treatment (Fetoni et al., 2016; Frisina et al., 2016; Gentilin et al., 2019). Oxidative stress injury is a major determinant of cell survival or death in cisplatin induced ototoxicity (Poirrier et al., 2010; Rybak et al., 2019; Waissbluth et al., 2012). NADPH oxidase 3 and xanthine oxidase are involved in the production of ROS in ototoxicity induced by cisplatin (Banfi et al., 2004; Lynch et al., 2005; Mukherjea et al., 2010) ) (Figure 2). Mitochondria play an important role in the regulation of cell apoptosis induced by intracellular stimulation (Bock and Tait, 2020), while oxidative stress is closely related to hair cell apoptosis through mitochondrial pathway (Karasawa and Steyger, 2015).

Cisplatin induces HEI-OC1 cell apoptosis, which is mainly manifested in nuclear concentration, DNA ladder and caspase-3 activation (Rybak et al., 1999). The decrease of intracellular ROS level after cisplatin application is the key to reduce the apoptosis and death of HEI-OC1 cells and cochlear hair cells caused by caspase 3 released by mitochondrial apoptosis. In previous studies, the total intracellular ROS and mitochondrial ROS change almost synchronously (Yang et al., 2018; Peoples et al., 2019). Interestingly, after Nrf2 activation, the total ROS level in hair cells decreases, whereas ROS level in mitochondria remains at a high level. In addition, after the combined action of cisplatin and Nrf2 activator, mitochondrial internal ROS in HEI-OC1 cells and cochlear hair cells remain unchanged during the activation of Nrf2, while the total intracellular ROS accumulation is significantly reduced (Zhang et al., 2020), suggesting that Nrf2 activation is not related to the production of mitochondrial internal ROS in the reduction of cisplatin induced oxidative stress and apoptosis. The interaction between intracellular and mitochondrial ROS levels needs to be further studied in the hearing impairment.

The nuclear translocation and activation of Nrf2 are related to the regulation of other signaling pathways. Mitogen-activated protein kinase (MAPK) and phosphatidylinositide 3-kinase (PI3K) signaling pathways are recognized to be involved in the nuclear translocation and activation of Nrf2 (Martin et al., 2004). However, in the correlation study of hearing impairment, only PI3K-Akt signaling pathway participated in the induction of Nrf2 nuclear translocation. The study has also found that Akt signaling is usually involved in the intracellular stimulation and activation of the natural product Nrf2 (Wang et al., 2012; Niture et al., 2014). Flunarizine and other antioxidative drugs can significantly increase the phosphorylation of Akt in a time-dependent manner and improve the nuclear translocation of Nrf2, while the decrease of the phosphorylation level of Akt significantly affects the expression of Nrf2. Moreover, PI3K-Akt signaling inhibitor can reduce the expression of Nrf2 and HO-1, suggesting that the Akt signaling pathway is involved in the regulation of Nrf2/HO-1, and the reduced phosphorylation level of Akt affects the expression of Nrf2 (So et al., 2006; So et al., 2008; Ma et al., 2015; Youn et al., 2017; Zhu et al., 2018).

Nrf2-ARE signaling pathway takes a prominent role in regulating phase II detoxifying and antioxidant genes. HO-1 is a downstream product of Nrf2, which plays an antioxidant, anti-apoptotic and anti-inflammatory role through its products bilirubin/biliverdin and carbon monoxide (Ma. 2013). HO-1 has been considered to be a critical antioxidant factor for resistance to cisplatin induced oxidative stress injury after activation of Nrf2-ARE signaling pathway in a number of studies (Ma et al., 2015; Youn et al., 2017). Antioxidant drugs can promote the nuclear translocation of Nrf2 in HCs and SGNs after cisplatin treatment, activate Nrf2-ARE signaling pathway, and improve the expression of antioxidant factors such as HO-1 (Fetoni et al., 2015). In the HEI-OC1 cell injury, cisplatin can significantly reduce the binding of HO-1 promoter to Nrf2. However, Ginkgolide B (GB) can significantly suppress the inhibitory effect of cisplatin on the binding of HO-1 promoter to Nrf2, and activate the Akt-Nrf2-HO-1 pathway to reduce the generation of ROS, thereby inhibiting mitochondrial apoptosis and ultimately reducing the toxicity induced by cisplatin (Ma et al., 2015). However, some studies have found that after the combined application of antioxidant drugs and cisplatin, HO-1 and SOD may not be the main components that play the role of hearing protection after the activation of Nrf2-ARE signaling pathway (Hoshino et al., 2011; Kim et al., 2015). Bucillamine induced translocation of Nrf2 to promote the expression of γ-GCS, GSS, GSH, HO-1 and SOD2. However, after gene knockout of HO-1 and SOD2, the protective effect of bucillamine on hearing seems to be independent of the enzyme activity of HO-1 and SOD (Kim et al., 2015). Another study also shows no significant difference in HO-1 expression between wild-type and Nrf2-KO mice after gentamicin exposure (Hoshino et al., 2011). Based on different research models or different experimental settings, there may be differences in the regulation of HO-1 expression by Nrf2. However, according to the current literature, HO-1 is indeed an important link for Nrf2 to play an antioxidant role.

Similar to the ototoxicity induced by cisplatin, ROS mediated oxidative stress loss is one of the main factors of aminoglycosides induced cochlear injury (Noack et al., 2017; Sekulic-Jablanovic et al., 2017). The aminoglycoside iron complex and aminoglycoside drug-induced mitochondrial dysfunction are important causes of intracellular ROS production. Sestrin-2 (Sesn2) is a member of the oxidative stress pathway, and the loss of Sesn2 increases the susceptibility of hair cells to gentamicin. Sesn2 may counteract the toxic effects of gentamicin on hair cells by activating Nrf2(Bodmer and Levano-Huaman, 2017). In Nrf2-knockout (KO) and wild-type mice, Nrf2 protects hair cells in wild-type mice from gentamicin damage by up-regulating antioxidant enzymes (Honkura et al., 2016).

It is worth mentioning that there is a positive feedback relationship between autophagy and Keap1/Nrf2 signaling pathway (Figure 2). Autophagy plays a pivotal role as an intracellular clearance system, cross-talking with Keap1/Nrf2 signaling pathway (Taguchi et al., 2011; Tang et al., 2019). p62 is a cohesive protein and plays an important role in autophagy as a molecular hub (Kirkin et al., 2009). p62 interacts directly with Keap1 and disrupts the association between Keap1 and Nrf2, thereby enhancing Nrf2 stability and nuclear accumulation (Komatsu et al., 2010). Moreover, the p62 gene is the target of Nrf2, and the accumulation of Nrf2 promotes the expression of p62 (Ishii et al., 2000). p62 alleviates H_2_O_2_-induced HEI-OC1 cell damage by promoting autophagy and activation of Keap1/Nrf2 signaling pathway, suggesting that p62 has the potential to serve as an adaptor protein between autophagy and the Keap1/Nrf2 signaling pathway in auditory cells (Copple et al., 2010; Hayashi et al., 2015).

## Drugs Acting on Nrf2 for Hearing Protection

Research on hearing protection targeting Nrf2 is ongoing, however, this research is mainly executed on animals and cells *in vitro*. In light of this, there is still a long way to go approach clinical application. In cell or animal experiments involving Nrf2, the hearing protection effect of various drugs is mainly realized by promoting the intracellular translocation of Nrf2 and the increase of its downstream target gene products, which we have listed in Table 1 and Table 2.

**TABLE 1:** Drugs acting on Nrf2 for hearing protection *in vitro* studies.

Agents	Function	Model systems	References
THSG	Activation of Nrf2 nuclear translocation and increase of HO-1 and NQO1	The mouse cochlear UB/OC-2 cell line	Wu T. Y. et al. (2020)
MIF	Activator of Akt-Nrf2-HO-1signaling pathway and increase of HO-1 and NQO1	HEI-OC1 cells	Zhu et al. (2018)
Flunarizine	Induction of Nrf2 nuclear translocation by PI3K/Akt signaling pathway and increase of HO-1	HEI-OC1 cells and corti organ	So et al. (2006)
			So et al. (2008)
11R-VIVIT	Promotion of Nrf2 transcription and increase of HO-1	C57BL/6N mice organ of corti explants	Sekulic-Jablanovic et al. (2020)
Phloretin	Activation of Nrf2 and JNK signaling pathways and induction of HO-1 expression	HEI-OC1 cells	Choi et al. (2011)
Piperine	Increase of Nrf2 nuclear translocation	HEI-OC1 cells	Choi et al. (2007)
Rapamycin	Increased Nrf2 expression via autophagy activation	HEI-OC1 cells	Hayashi et al. (2015)

**TABLE 2:** Drugs acting on Nrf2 for hearing protection *in vitro* studies.

Agents	Function	Model systems	References
Curcumin	Activation of Nrf2 nuclear translocation and enhance endogenous antioxidant capacity	Wistar rats	Fetoni et al. (2015a)
Curcumin and ferulic acid	Activation of Nrf2 nuclear translocation and increase of HO-1 in HCs and SGNs	Wistar rats	Paciello et al. (2020)
Bucillamine	Induction of Nrf2 nuclear translocation and increase of γ-GCS,GSS,GSH,HO-1and SOD2	Balb/C male mice and HEI-OC1 cells	Kim et al. (2015)
Rosmarinic acid	Induction of Nrf2 nuclear translocation and increase of HO-1 and SOD	Wistar rats	Fetoni et al. (2015b)
CDDO-Im	Activation of Nrf2 and increase of NQO1, HO-1, GCLC, GCLM and Txnrd1	Wild-type and Nrf2-KO C57BL/6 mice	Honkura et al. (2016)
Ginkgolide B	Reduction of intracelluar ROS by activation of Akt-Nrf2-HO-1 signaling pathway	HEI-OC1 cells and SD rats	Ma et al. (2015)
TBHQ	Inhibition of intracellular ROS outside mitochondria by activation of Nrf2	HEI−OC1 cell and C57BL/6 mice	Zhang et al. (2020)
Ferulic acid	Reduction of intracelluar ROS by increase of Nrf2	HEI-OC1 cells and C57BL/6 mice	Jo et al. (2019)
Interleukin-10	Inhibition of NF-κB signaling pathway by activation of Nrf2/CO-mediated feedback loop	C57BL/6 mice and rat spiral ligament fibrocyte cell line	Mwangi et al. (2017)
Sulforaphane	Enhancement of ERK activation and possibly further regulation of Nrf2 expression	Tub/tub mice and tub/WT mice	Kong et al. (2009)
Ebselen	Activation of Nrf2 and increase of HO-1, NADPH and γ-GCS	HEI-OC1 cells and Balb/C mice	Kim et al. (2009)

As far as the current *in vivo* experiments are concerned, the research on the role of Nrf2 in protecting hair cells seems only heading in one direction, showing a single mode of activating Nrf2 after drug administration, promoting the expression of its downstream genes and protecting hair cells from damage.

Although the interactions between Nrf2 and its downstream gene products and other transcription factors were considered in some studies, the key molecules are still far from enough to fully reveal the complete network involved in Nrf2 regulation after drug administration. In these *in vitro* studies, although the differences in the protective effects of different dosages of drugs on hearing loss were explored in some settings and the dose-response curves were plotted, future *in vitro* studies are indispensable to determine the maximum amount of drugs and the resulting acute and chronic toxicity, including pathological sections of major organs after administration. Moreover, the best route, time and dosage of drug administration need to be further optimized. Ebselen, an old analgesic and anti-inflammatory drug, has been shown to reduce oxidative stress damage in hair cells induced by cisplatin via activating the Nrf2-ARE signaling pathway and increasing the expression of HO-1, NADPH and γ-GCS(Kim et al., 2009). In addition, ebselen has been shown to prevent and protect against hearing loss caused by noise and aminoglycosides in animal studies (Kil et al., 2007; Gu et al., 2021). In clinical trials, oral administration of ebselen, 400 mg twice daily, can safely and effectively prevent noise induced temporary threshold shift (TTS) (Kil et al., 2017). At present, ebselen is in phase IIb clinical trial, and the feasibility of the drug acting on Nrf2 to prevent and treat hearing loss deserves our expectation.

## Prospects

Nrf2 has been proved as an important target for the treatment of neurodegenerative diseases, respiratory diseases, digestive diseases, cardiovascular diseases, metabolic diseases and cardiovascular diseases. Meanwhile, a variety of drugs have also been discovered to possess antioxidant effects by regulating the expression of Nrf2. The etiology of hearing loss is complex and unclear. Reducing hearing loss caused by oxidative stress injury is considered as one of the greatest challenges. This review summarized the current studies on the role of Nrf2 in various subtypes of sensorineural hearing loss, and lists the current drug studies that act on Nrf2 for hearing protection or to protect HEI-OC1 cell from injury. However, how Nrf2 regulates ROS changes in hair cells and the upstream and downstream regulatory network of Nrf2 in hair cells are still not fully understood. Studies on the early prevention and treatment of hearing loss through the Keap1-Nrf2-ARE signaling axis are still at the exploratory stage, although the new and effective Nrf2 inducer has been proven effective for hearing protection in animal models. In addition, the safety of Nrf2 inducers or activators also merits more attention.

There is an urgent need to clarify the following issues. First, the interactions of Nrf2 with other transcription factors should be further investigated to obtain a network for related drug discovery. Second, the development of better cell, animal models which support a real reflection of the pathological conditions of hearing loss. Third, pre-clinical experiments should be executed deliberately to acquire optimized results for transplantation into clinical trials.

## Glossary

ABR: auditory brainstem response
ARE: antioxidant response element
ARHL: age-related hearing loss
bZIP: basic region-Leucine Zipper
CDDO-Im: 2-cyano-3,12 dioxooleana-1,9 dien-28-imidazolide
CRISPR: clustered regularly interspaced short palindromic repeat
Cul3: cullin3
Cx26: connexin 26
ERK: extracellular signal-regulated kinase
GB: ginkgolide B
GJB2: gap junction protein beta-2
GCLC: glutamate cysteine ligase catalytic
*γ*-GCS: *γ*-glutamyl cysteine synthetase
GSS: glutathione synthase;
GSH: glutathione
GSTs: glutathione-S-transferses
HO-1: heme oxygenase-1
IHC: inner hair cell
IR: immunoreactivity
Keap1: Kelch-like ECH-Associating protein 1
KO: knockout
MAPK: mitogen-activated protein kinase
MIF: macrophage migration inhibitory factor
NADPH: nicotinamide adenine dinucleotide phosphate
NIHL: noise induced hearing loss
NQO1: quinone oxidoreductase 1
Nrf2: nuclear factor erythroid 2-related factor 2
OHC: outer hair cell
PI3K: phosphatidylinositide 3-kinase
PRKCE: Protein Kinase C Epsilon
ROS: reactive oxygen species
RNS: reactive nitrogen species
Sesn2: sestrin-2
SGNs: spiral ganglion neurons
sMafs: small Maf proteins
SNHL: sensorineural hearing loss
SNP: single nucleotide polymorphism
SOD2: superoxide dismutase 2
SODs: superoxide dismutases
TBHQ: tert-butylhydroquinone
TGF-β1: transforming growth factor-β1
THSG: 2,3,4’,5-Tetrahydroxystilbene-2-O-β-D-Glucoside
TTS: temporary threshold shift
Txnrd1: Thioredoxin reductase 1

## References

[B1] Baird L. Dinkova-Kostova A. T. (2011). The cytoprotective role of the Keap1-Nrf2 pathway. Arch. Toxicol. 85, 241–272. 10.1007/s00204-011-0674-5 21365312

[B2] Bánfi B. Malgrange B. Knisz J. Steger K. Dubois-Dauphin M. Krause K.-H. (2004). NOX3, a superoxide-generating NADPH oxidase of the inner ear. J. Biol. Chem. 279, 46065–46072. 10.1074/jbc.M403046200 15326186

[B3] Bock F. J. Tait S. W. G. (2020). Mitochondria as multifaceted regulators of cell death. Nat. Rev. Mol. Cel Biol 21, 85–100. 10.1038/s41580-019-0173-8 31636403

[B4] Bodmer D. Levano-Huaman S. (2017). Sesn2/AMPK/mTOR signaling mediates balance between survival and apoptosis in sensory hair cells under stress. Cell Death Dis 8, e3068. 10.1038/cddis.2017.457 28981119 PMC5680579

[B5] Brock P. R. Maibach R. Neuwelt E. A. (2018). Sodium thiosulfate and cisplatin-induced hearing. Loss N. Engl. J. Med. 379, 1181. 10.1056/NEJMc1809501 30231227

[B6] Choi B. M. Chen X. Y. Gao S. S. Zhu R. Kim B. R. (2011). Anti-apoptotic effect of phloretin on cisplatin-induced apoptosis in HEI-OC1 auditory cells. Pharmacol. Rep. 63, 708–716. 10.1016/s1734-1140(11)70582-5 21857081

[B7] Choi B. M. Kim S. M. Park T. K. Li G. Hong S. J. Park R. (2007). Piperine protects cisplatin-induced apoptosis via heme oxygenase-1 induction in auditory cells. J. Nutr. Biochem. 18, 615–622. 10.1016/j.jnutbio.2006.11.012 17418561

[B8] Copple I. M. Lister A. Obeng A. D. Kitteringham N. R. Jenkins R. E. Layfield R. (2010). Physical and functional interaction of sequestosome 1 with Keap1 regulates the Keap1-Nrf2 cell defense pathway. J. Biol. Chem. 285, 16782–16788. 10.1074/jbc.M109.096545 20378532 PMC2878012

[B9] Cuadrado A. Manda G. Hassan A. Alcaraz M. J. Barbas C. Daiber A. (2018). Transcription factor NRF2 as a therapeutic target for chronic diseases: a systems medicine approach. Pharmacol. Rev. 70, 348–383. 10.1124/pr.117.014753 29507103

[B10] Cuadrado A. Pajares M. Benito C. Jiménez-Villegas J. Escoll M. Fernández-Ginés R. (2020). Can activation of NRF2 Be a strategy against COVID-19?. Trends Pharmacol. Sci. 41, 598–610. 10.1016/j.tips.2020.07.003 32711925 PMC7359808

[B11] Cuadrado A. Rojo A. I. Wells G. Hayes J. D. Cousin S. P. Rumsey W. L. (2019). Therapeutic targeting of the NRF2 and KEAP1 partnership in chronic diseases. Nat. Rev. Drug Discov. 18, 295–317. 10.1038/s41573-018-0008-x 30610225

[B12] Cunningham L. L. Tucci D. L. (2017). Hearing loss in adults. N. Engl. J. Med. 377, 2465–2473. 10.1056/NEJMra1616601 29262274 PMC6457651

[B13] Fão L. Mota S. I. Rego A. C. (2019). Shaping the Nrf2-ARE-related pathways in Alzheimer's and Parkinson's diseases. Ageing Res. Rev. 54, 100942. 10.1016/j.arr.2019.100942 31415806

[B14] Fetoni A. R. Ruggiero A. Lucidi D. De Corso E. Sergi B. Conti G. (2016). Audiological monitoring in children treated with platinum chemotherapy. Audiol. Neurootol. 21, 203–211. 10.1159/000442435 27286730

[B15] Fetoni A. R. Paciello F. Mezzogori D. Rolesi R. Eramo S. L. M. Paludetti G. (2015a). Molecular targets for anticancer redox chemotherapy and cisplatin-induced ototoxicity: the role of curcumin on pSTAT3 and Nrf-2 signalling. Br. J. Cancer 113, 1434–1444. 10.1038/bjc.2015.359 26469832 PMC4815880

[B16] Fetoni A. R. Paciello F. Rolesi R. Eramo S. L. M. Mancuso C. Troiani D. (2015b). Rosmarinic acid up-regulates the noise-activated Nrf2/HO-1 pathway and protects against noise-induced injury in rat cochlea. Free Radic. Biol. Med. 85, 269–281. 10.1016/j.freeradbiomed.2015.04.021 25936352

[B17] Fetoni A. R. Paciello F. Rolesi R. Paludetti G. Troiani D. (2019). Targeting dysregulation of redox homeostasis in noise-induced hearing loss: oxidative stress and ROS signaling. Free Radic. Biol. Med. 135, 46–59. 10.1016/j.freeradbiomed.2019.02.022 30802489

[B18] Fetoni A. R. Zorzi V. Paciello F. Ziraldo G. Peres C. Raspa M. (2018). Cx26 partial loss causes accelerated presbycusis by redox imbalance and dysregulation of Nfr2 pathway. Redox Biol. 19, 301–317. 10.1016/j.redox.2018.08.002 30199819 PMC6129666

[B19] Franzé A. Caravelli A. Leva F. Marciano E. Auletta G. D’Aulos F. (2005). Audiometric evaluation of carriers of the connexin 26 mutation 35delG. Eur. Arch. Otorhinolaryngol. 262, 921–924. 10.1007/s00405-005-0918-1 15895291

[B20] Frisina R. D. Wheeler H. E. Fossa S. D. Kerns S. L. Fung C. Sesso H. D. (2016). Comprehensive audiometric analysis of hearing impairment and tinnitus after cisplatin-based chemotherapy in survivors of adult-onset cancer. J. Clin. Oncol. 34, 2712–2720. 10.1200/JCO.2016.66.8822 27354478 PMC5019759

[B21] Fujimoto C. Yamasoba T. (2019). Mitochondria-targeted antioxidants for treatment of hearing loss: a systematic review. Antioxidants 8, 109. 10.3390/antiox8040109 PMC6523236 31022870

[B22] Gentilin E. Simoni E. Candito M. Cazzador D. Astolfi L. (2019). Cisplatin-induced ototoxicity: updates on molecular targets. Trends Mol. Med. 25, 1123–1132. 10.1016/j.molmed.2019.08.002 31473143

[B23] Gu R. Longenecker R. J. Homan J. Kil J. (2020). Ebselen attenuates tobramycin-induced ototoxicity in mice. J. Cyst Fibros. 20, 271-277. 10.1016/j.jcf.2020.02.014 32147183

[B24] Hayashi K. Dan K. Goto F. Tshuchihashi N. Nomura Y. Fujioka M. (2015). The autophagy pathway maintained signaling crosstalk with the Keap1-Nrf2 system through p62 in auditory cells under oxidative stress. Cell Signal. 27, 382–393. 10.1016/j.cellsig.2014.11.024 25435427

[B25] Henderson D. Bielefeld E. C. Harris K. C. Hu B. H. (2006). The role of oxidative stress in noise-induced hearing loss. Ear Hear 27, 1–19. 10.1097/01.aud.0000191942.36672.f3 16446561

[B26] Honkura Y. Matsuo H. Murakami S. Sakiyama M. Mizutari K. Shiotani A. (2016). NRF2 is a key target for prevention of noise-induced hearing loss by reducing oxidative damage of cochlea. Sci. Rep. 6, 19329. 10.1038/srep19329 26776972 PMC4726010

[B27] Hoshino T. Tabuchi K. Nishimura B. Tanaka S. Nakayama M. Ishii T. (2011). Protective role of Nrf2 in age-related hearing loss and gentamicin ototoxicity. Biochem. Biophys. Res. Commun. 415, 94–98. 10.1016/j.bbrc.2011.10.019 22020098

[B28] Hosokawa K. Hosokawa S. Ishiyama G. Ishiyama A. Lopez I. A. (2018). Immunohistochemical localization of Nrf2 in the human cochlea. Brain Res. 1700, 1–8. 10.1016/j.brainres.2018.07.004 29981724 PMC6231984

[B29] Igarashi K. Kataokat K. Itoh K. Hayashi N. Nishizawa M. Yamamoto M. (1994). Regulation of transcription by dimerization of erythroid factor NF-E2 p45 with small Maf proteins. Nature 367, 568–572. 10.1038/367568a0 8107826

[B30] Ishii T. Itoh K. Takahashi S. Sato H. Yanagawa T. Katoh Y. (2000). Transcription factor Nrf2 coordinately regulates a group of oxidative stress-inducible genes in macrophages. J. Biol. Chem. 275, 16023–16029. 10.1074/jbc.275.21.16023 10821856

[B31] Itoh K. Tong K. I. Yamamoto M. (2004). Molecular mechanism activating nrf2-keap1 pathway in regulation of adaptive response to electrophiles. Free Radic. Biol. Med. 36, 1208–1213. 10.1016/j.freeradbiomed.2004.02.075 15110385

[B32] Jaramillo M. C. Zhang D. D. (2013). The emerging role of the Nrf2-Keap1 signaling pathway in cancer. Genes Dev. 27, 2179–2191. 10.1101/gad.225680.113 24142871 PMC3814639

[B33] Jo E.-R. Youn C. K. Jun Y. Cho S. I. (2019). The protective role of ferulic acid against cisplatin-induced ototoxicity. Int. J. Pediatr. Otorhinolaryngol. 120, 30–35. 10.1016/j.ijporl.2019.02.001 30753979

[B34] Jung K. A. Kwak M. K. (2010). The Nrf2 system as a potential target for the development of indirect antioxidants. Molecules 15, 7266–7291. 10.3390/molecules15107266 20966874 PMC6259123

[B35] Karasawa T. Steyger P. S. (2015). An integrated view of cisplatin-induced nephrotoxicity and ototoxicity. Toxicol. Lett. 237, 219–227. 10.1016/j.toxlet.2015.06.012 26101797 PMC4516600

[B36] Kataoka K. Igarashi K. Itoh K. Fujiwara K. T. Noda M. Yamamoto M. (1995). Small Maf proteins heterodimerize with Fos and may act as competitive repressors of the NF-E2 transcription factor. Mol. Cel. Biol. 15, 2180–2190. 10.1128/mcb.15.4.2180 PMC230446 7891713

[B37] Katsuoka F. Yamamoto M. (2016). Small Maf proteins (MafF, MafG, MafK): history, structure and function. Gene 586, 197–205. 10.1016/j.gene.2016.03.058 27058431 PMC4911266

[B38] Kil J. Lobarinas E. Spankovich C. Griffiths S. K. Antonelli P. J. Lynch E. D. (2017). Safety and efficacy of ebselen for the prevention of noise-induced hearing loss: a randomised, double-blind, placebo-controlled, phase 2 trial. Lancet 390, 969–979. 10.1016/S0140-6736(17)31791-9 28716314

[B39] Kil J. Pierce C. Tran H. Gu R. Lynch E. D. (2007). Ebselen treatment reduces noise induced hearing loss via the mimicry and induction of glutathione peroxidase. Hearing Res. 226, 44–51. 10.1016/j.heares.2006.08.006 17030476

[B40] Kim S. J. Ho Hur J. Park C. Kim H. J. Oh G. S. Lee J. N. (2015). Bucillamine prevents cisplatin-induced ototoxicity through induction of glutathione and antioxidant genes. Exp. Mol. Med. 47, e142. 10.1038/emm.2014.112 25697147 PMC4346486

[B41] Kim S. J. Park C. Han A. L. Youn M. J. Lee J. H. Kim Y. (2009). Ebselen attenuates cisplatin-induced ROS generation through Nrf2 activation in auditory cells. Hearing Res. 251, 70–82. 10.1016/j.heares.2009.03.003 19286452

[B42] Kirkin V. McEwan D. G. Novak I. Dikic I. (2009). A role for ubiquitin in selective autophagy. Mol. Cel 34, 259–269. 10.1016/j.molcel.2009.04.026 19450525

[B43] Kobayashi A. Ito E. Toki T. Kogame K. Takahashi S. Igarashi K. (1999). Molecular cloning and functional characterization of a new Cap'n' collar family transcription factor Nrf3. J. Biol. Chem. 274, 6443–6452. 10.1074/jbc.274.10.6443 10037736

[B44] Komatsu M. Kurokawa H. Waguri S. Taguchi K. Kobayashi A. Ichimura Y. (2010). The selective autophagy substrate p62 activates the stress responsive transcription factor Nrf2 through inactivation of Keap1. Nat. Cel Biol 12, 213–223. 10.1038/ncb2021 20173742

[B45] Kong L. Chen G. D. Zhou X. McGinnis J. F. Li F. Cao W. (2009). Molecular mechanisms underlying cochlear degeneration in the tubby mouse and the therapeutic effect of sulforaphane. Neurochem. Int. 54, 172–179. 10.1016/j.neuint.2008.08.013 19114066 PMC2689618

[B46] Kros C. J. Steyger P. S. (2019). Aminoglycoside- and cisplatin-induced ototoxicity: mechanisms and otoprotective strategies. Cold Spring Harb Perspect. Med. 9, a033548. 10.1101/cshperspect.a033548 30559254 PMC6579718

[B47] Liu X. Yan D. (2007). Ageing and hearing loss. J. Pathol. 211, 188–197. 10.1002/path.2102 17200945

[B48] Liu Y. Lang F. Yang C. (2020). NRF2 in human neoplasm: cancer biology and potential therapeutic target. Pharmacol Ther. 217, 107664. 10.1016/j.pharmthera.2020.107664 32810525

[B49] Lynch E. D. Gu R. Pierce C. Kil J. (2005). Reduction of acute cisplatin ototoxicity and nephrotoxicity in rats by oral administration of allopurinol and ebselen. Hearing Res. 201, 81–89. 10.1016/j.heares.2004.08.002 15721563

[B50] Ma Q. (2013). Role of nrf2 in oxidative stress and toxicity. Annu. Rev. Pharmacol. Toxicol. 53, 401–426. 10.1146/annurev-pharmtox-011112-140320 23294312 PMC4680839

[B51] Ma W. Hu J. Cheng Y. Wang J. Zhang X. Xu M. (2015). Ginkgolide B protects against cisplatin-induced ototoxicity: enhancement of Akt-Nrf2-HO-1 signaling and reduction of NADPH oxidase. Cancer Chemother. Pharmacol. 75, 949–959. 10.1007/s00280-015-2716-9 25749575

[B52] Martin D. Rojo A. I. Salinas M. Diaz R. Gallardo G. Alam J. (2004). Regulation of heme oxygenase-1 expression through the phosphatidylinositol 3-kinase/Akt pathway and the Nrf2 transcription factor in response to the antioxidant phytochemical carnosol. J. Biol. Chem. 279, 8919–8929. 10.1074/jbc.M309660200 14688281

[B53] Marx M. Younes E. Chandrasekhar S. S. Ito J. Plontke S. O’Leary S. (2018). International consensus (ICON) on treatment of sudden sensorineural hearing loss. Eur. Ann. Otorhinolaryngol. Head Neck Dis. 135, S23–S28. 10.1016/j.anorl.2017.12.011 29396226

[B54] Maulucci G. Troiani D. Eramo S. L. M. Paciello F. Podda M. V. Paludetti G. (2014). Time evolution of noise induced oxidation in outer hair cells: role of NAD(P)H and plasma membrane fluidity. Biochim. Biophys. Acta (Bba) - Gen. Subjects 1840, 2192–2202. 10.1016/j.bbagen.2014.04.005 24735797

[B55] Menardo J. Tang Y. Ladrech S. Lenoir M. Casas F. Michel C. (2012). Oxidative stress, inflammation, and autophagic stress as the key mechanisms of premature age-related hearing loss in SAMP8 mouse cochlea. Antioxid. Redox Signaling 16, 263–274. 10.1089/ars.2011.4037 21923553

[B56] Milkovic L. Zarkovic N. Saso L. (2017). Controversy about pharmacological modulation of Nrf2 for cancer therapy. Redox Biol. 12, 727–732. 10.1016/j.redox.2017.04.013 28411557 PMC5393166

[B57] Mukherjea D. Jajoo S. Kaur T. Sheehan K. E. Ramkumar V. Rybak L. P. (2010). Transtympanic administration of short interfering (si)RNA for the NOX3 isoform of NADPH oxidase protects against cisplatin-induced hearing loss in the rat. Antioxid. Redox Signaling 13, 589–598. 10.1089/ars.2010.3110 PMC2935347 20214492

[B58] Mwangi M. Kil S.-H. Phak D. Park H. Y. Lim D. J. Park R. (2017). Interleukin-10 attenuates hypochlorous acid-mediated cytotoxicity to HEI-OC1 cochlear cells. Front. Cel. Neurosci. 11, 314. 10.3389/fncel.2017.00314 PMC5635053 29056901

[B59] Niture S. K. Khatri R. Jaiswal A. K. (2014). Regulation of nrf2-an update. Free Radic. Biol. Med. 66, 36–44. 10.1016/j.freeradbiomed.2013.02.008 23434765 PMC3773280

[B60] Noack V. Pak K. Jalota R. Kurabi A. Ryan A. F. (2017). An antioxidant screen identifies candidates for protection of cochlear hair cells from gentamicin toxicity. Front. Cell. Neurosci. 11, 242. 10.3389/fncel.2017.00242 28867994 PMC5563352

[B61] Ohlemiller K. K. Hughes R. M. Mosinger-Ogilvie J. Speck J. D. Grosof D. H. Silverman M. S. (1995). Cochlear and retinal degeneration in the tubby mouse. Neuroreport 6, 845–849. 10.1097/00001756-199504190-00005 7612867

[B62] Ohlemiller K. K. Wright J. S. Dugan L. L. (1999). Early elevation of cochlear reactive oxygen species following noise exposure. Audiol. Neurootol. 4, 229–236. 10.1159/000013846 10436315

[B63] Oyake T. Itoh K. Motohashi H. Hayashi N. Hoshino H. Nishizawa M. (1996). Bach proteins belong to a novel family of BTB-basic leucine zipper transcription factors that interact with MafK and regulate transcription through the NF-E2 site. Mol. Cel. Biol. 16, 6083–6095. 10.1128/mcb.16.11.6083 PMC231611 8887638

[B64] Paciello F. Fetoni A. R. Mezzogori D. Rolesi R. Di Pino A. Paludetti G. (2020). The dual role of curcumin and ferulic acid in counteracting chemoresistance and cisplatin-induced ototoxicity. Sci. Rep. 10, 1063. 10.1038/s41598-020-57965-0 31974389 PMC6978317

[B65] Panieri E. Buha A. Telkoparan-Akillilar P. Kouretas D. Veskoukis A. Skaperda Z. (2020). Potential applications of NRF2 modulators in cancer therapy. Antioxidants (Basel) 9, 193. 10.3390/antiox9030193 PMC7139512 32106613

[B66] Peoples J. N. Saraf A. Ghazal N. Pham T. T. Kwong J. Q. (2019). Mitochondrial dysfunction and oxidative stress in heart disease. Exp. Mol. Med. 51, 1–13. 10.1038/s12276-019-0355-7 PMC6923355 31857574

[B67] Poirrier A. L. Pincemail J. Van Den Ackerveken P. Lefebvre P. P. Malgrange B. (2010). Oxidative stress in the cochlea: an update. Curr. Med. Chem. 17, 3591–3604. 10.2174/092986710792927895 20738243

[B68] Prestera T. Zhang Y. Spencer S. R. Wilczak C. A. Talalay P. (1993). The electrophile counterattack response: protection against neoplasia and toxicity. Adv. Enzyme Regul. 33, 281–296. 10.1016/0065-2571(93)90024-8 8356913

[B69] Quirk W. S. Seidman M. D. (1995). Cochlear vascular changes in response to loud noise. Am. J. Otol 16, 322–325. 8588626

[B70] Quirk W. S. Avinash G. Nuttall A. L. Miller J. M. (1992). The influence of loud sound on red blood cell velocity and blood vessel diameter in the cochlea. Hearing Res. 63, 102–107. 10.1016/0378-5955(92)90079-3 1464564

[B71] Rousset F. Nacher-Soler G. Coelho M. Ilmjarv S. Kokje V. B. C. Marteyn A. (2020). Redox activation of excitatory pathways in auditory neurons as mechanism of age-related hearing loss. Redox Biol. 30, 101434. 10.1016/j.redox.2020.101434 32000019 PMC7016250

[B72] Rybak L. Mukherjea D. Ramkumar V. (2019). Mechanisms of cisplatin-induced ototoxicity and prevention. Semin. Hear. 40, 197–204. 10.1055/s-0039-1684048 31036996 PMC6486366

[B73] Rybak L. P. Whitworth C. Somani S. (1999). Application of antioxidants and other agents to prevent cisplatin ototoxicity. Laryngoscope 109, 1740–1744. 10.1097/00005537-199911000-00003 10569399

[B74] Satoh T. Akhtar M. W. Lipton S. A. (2013). “Combating oxidative/nitrosative stress with electrophilic counterattack strategies,” in Oxidative stress and redox regulation. Editors Jakob U. Reichmann D. (Dordrecht, Netherlands: Springer), 277–307. 10.1007/978-94-007-5787-5_10

[B75] Sekulic-Jablanovic M. Voronkova K. Bodmer D. Petkovic V. (2020). Combination of antioxidants and NFAT (nuclear factor of activated T cells) inhibitor protects auditory hair cells from ototoxic insult. J. Neurochem. 154, 519–529. 10.1111/jnc.14921 31755556

[B76] Sekulic-Jablanovic M. Petkovic V. Wright M. B. Kucharava K. Huerzeler N. Levano S. (2017). Effects of peroxisome proliferator activated receptors (PPAR)-γ and -α agonists on cochlear protection from oxidative stress. PLoS One 12, e0188596. 10.1371/journal.pone.0188596 29182629 PMC5705132

[B77] So H. S. Kim H. J. Lee J. H. Lee J. H. Park S. Y. Park C. (2006). Flunarizine induces Nrf2-mediated transcriptional activation of heme oxygenase-1 in protection of auditory cells from cisplatin. Cell Death Differ 13, 1763–1775. 10.1038/sj.cdd.4401863 16485034

[B78] So H. Kim H. Kim Y. Kim E. Pae H. O. Chung H. T. (2008). Evidence that cisplatin-induced auditory damage is attenuated by downregulation of pro-inflammatory cytokines via Nrf2/HO-1. J. Assoc. Res. Otolaryngol 9, 290–306. 10.1007/s10162-008-0126-y 18584244 PMC2538144

[B79] Someya S. Yu W. Hallows W. C. Xu J. Vann J. M. Leeuwenburgh C. (2010). Sirt3 mediates reduction of oxidative damage and prevention of age-related hearing loss under caloric restriction. Cell 143, 802–812. 10.1016/j.cell.2010.10.002 21094524 PMC3018849

[B80] Someya S. Prolla T. A. (2010). Mitochondrial oxidative damage and apoptosis in age-related hearing loss. Mech. Ageing Dev. 131, 480–486. 10.1016/j.mad.2010.04.006 20434479 PMC4086639

[B81] Suzuki T. Shibata T. Takaya K. Shiraishi K. Kohno T. Kunitoh H. (2013). Regulatory nexus of synthesis and degradation deciphers cellular Nrf2 expression levels. Mol. Cell Biol. 33, 2402–2412. 10.1128/MCB.00065-13 23572560 PMC3700104

[B82] Sykiotis G. P. Bohmann D. (2010). Stress-activated cap'n'collar transcription factors in aging and human disease. Sci. Signaling 3, re3. 10.1126/scisignal.3112re3 PMC2991085 20215646

[B83] Taguchi K. Motohashi H. Yamamoto M. (2011). Molecular mechanisms of the Keap1-Nrf2 pathway in stress response and cancer evolution. Genes Cells 16, 123–140. 10.1111/j.1365-2443.2010.01473.x 21251164

[B84] Tang Z. Hu B. Zang F. Wang J. Zhang X. Chen H. (2019). Nrf2 drives oxidative stress-induced autophagy in nucleus pulposus cells via a Keap1/Nrf2/p62 feedback loop to protect intervertebral disc from degeneration. Cell Death Dis. 10, 510. 10.1038/s41419-019-1701-3 31263165 PMC6602960

[B85] Waissbluth S. Pitaro J. Daniel S. J. (2012). Gene therapy for cisplatin-induced ototoxicity: a systematic review of *in vitro* and experimental animal studies. Otol Neurotol 33, 302–310. 10.1097/MAO.0b013e318248ee66 22388732

[B86] Wang H. Liu K. Geng M. Gao P. Wu X. Hai Y. (2013). RXRα inhibits the NRF2-ARE signaling pathway through a direct interaction with the Neh7 domain of NRF2. Cancer Res. 73, 3097–3108. 10.1158/0008-5472.CAN-12-3386 23612120

[B87] Wang X. Wu H. Chen H. Liu R. Liu J. Zhang T. (2012). Does insulin bolster antioxidant defenses via the extracellular signal-regulated kinases-protein kinase B-nuclear factor erythroid 2 p45-related factor 2 pathway?. Antioxid. Redox Signaling 16, 1061–1070. 10.1089/ars.2011.4460 22149292

[B88] Williamson T. P. Johnson D. A. Johnson J. A. (2012). Activation of the Nrf2-ARE pathway by siRNA knockdown of Keap1 reduces oxidative stress and provides partial protection from MPTP-mediated neurotoxicity. Neurotoxicology 33, 272–279. 10.1016/j.neuro.2012.01.015 22342405 PMC3521526

[B89] Wilson B. S. Tucci D. L. Merson M. H. O'Donoghue G. M. (2017). Global hearing health care: new findings and perspectives. Lancet 390, 2503–2515. 10.1016/S0140-6736(17)31073-5 28705460

[B90] Wu F. Xiong H. Sha S. (2020). Noise-induced loss of sensory hair cells is mediated by ROS/AMPKα pathway. Redox Biol. 29, 101406. 10.1016/j.redox.2019.101406 31926629 PMC6933152

[B91] Wu T. Y. Lin J. N. Luo Z. Y. Hsu C. J. Wang J. S. Wu H. P. (2020). 2,3,4',5-Tetrahydroxystilbene-2-O-beta-D-Glucoside (THSG) activates the Nrf2 antioxidant pathway and attenuates oxidative stress-induced cell death in mouse cochlear UB/OC-2 cells. Biomolecules 10, 465. 10.3390/biom10030465 PMC7175305 32197448

[B92] Yamamoto T. Yoh K. Kobayashi A. Ishii Y. Kure S. Koyama A. (2004). Identification of polymorphisms in the promoter region of the human NRF2 gene. Biochem. Biophys. Res. Commun. 321, 72–79. 10.1016/j.bbrc.2004.06.112 15358217

[B93] Yamane H. Nakai Y. Konishi K. Sakamoto H. Matsuda Y. Iguchi H. (1991). Strial circulation impairment due to acoustic trauma. Acta Oto-Laryngol. 111, 85–93. 10.3109/00016489109137358 2014760

[B94] Yamashita D. Jiang H. Y. Le Prell C. G. Schacht J. Miller J. M. (2005). Post-exposure treatment attenuates noise-induced hearing loss. Neurosci. 134, 633–642. 10.1016/j.neuroscience.2005.04.015 15961244

[B95] Yamasoba T. Lin F. R. Someya S. Kashio A. Sakamoto T. Kondo K. (2013). Current concepts in age-related hearing loss: epidemiology and mechanistic pathways. Hearing Res. 303, 30–38. 10.1016/j.heares.2013.01.021 PMC3723756 23422312

[B96] Yamazaki H. Tanji K. Wakabayashi K. Matsuura S. Itoh K. (2015). Role of the Keap1/Nrf2 pathway in neurodegenerative diseases. Pathol. Int. 65, 210–219. 10.1111/pin.12261 25707882

[B97] Yang Q. Sun G. Yin H. Li H. Cao Z. Wang J. (2018). PINK1 protects auditory hair cells and spiral ganglion neurons from cisplatin-induced ototoxicity via inducing autophagy and inhibiting JNK signaling pathway. Free Radic. Biol. Med. 120, 342–355. 10.1016/j.freeradbiomed.2018.02.025 29458150

[B98] Youn C. K. Jo E. R. Sim J. H. Cho S. I. (2017). Peanut sprout extract attenuates cisplatin-induced ototoxicity by induction of the Akt/Nrf2-mediated redox pathway. Int. J. Pediatr. Otorhinolaryngol. 92, 61–66. 10.1016/j.ijporl.2016.11.004 28012535

[B99] Yuan H. Wang X. Hill K. Chen J. Lemasters J. Yang S. M. (2015). Autophagy attenuates noise-induced hearing loss by reducing oxidative stress. Antioxid. Redox Signal. 22, 1308–1324. 10.1089/ars.2014.6004 25694169 PMC4410759

[B100] Zada S. L. Baruch B. B. Simhaev L. Engel H. Fridman M. (2020). Chemical modifications reduce auditory cell damage induced by aminoglycoside antibiotics. J. Am. Chem. Soc. 142, 3077–3087. 10.1021/jacs.9b12420 31958945

[B101] Zhang W. Xiong H. Pang J. Su Z. Lai L. Lin H. (2020). Nrf2 activation protects auditory hair cells from cisplatin-induced ototoxicity independent on mitochondrial ROS production. Toxicol. Lett. 331, 1–10. 10.1016/j.toxlet.2020.04.005 32428544

[B102] Zhong Q. Mishra M. Kowluru R. A. (2013). Transcription factor Nrf2-mediated antioxidant defense system in the development of diabetic retinopathy. Invest. Ophthalmol. Vis. Sci. 54, 3941–3948. 10.1167/iovs.13-11598 23633659 PMC3676188

[B103] Zhu H. Jia Z. Misra B. R. Zhang L. Cao Z. Yamamoto M. (2008). Nuclear factor E2-related factor 2-dependent myocardiac cytoprotection against oxidative and electrophilic stress. Cardiovasc. Toxicol. 8, 71–85. 10.1007/s12012-008-9016-0 18463988

[B104] Zhu W. Y. Jin X. Ma Y. C. Liu Z. B. (2018). MIF protects against oxygen-glucose deprivation-induced ototoxicity in HEI-OC1 cochlear cells by enhancement of Akt-Nrf2-HO-1 pathway. Biochem. Biophys. Res. Commun. 503, 665–670. 10.1016/j.bbrc.2018.06.058 29908183

